# Survival Benefit of Living-Donor Liver Transplant

**DOI:** 10.1001/jamasurg.2022.3327

**Published:** 2022-08-03

**Authors:** Whitney E. Jackson, John S. Malamon, Bruce Kaplan, Jessica L. Saben, Jesse D. Schold, James J. Pomposelli, Elizabeth A. Pomfret

**Affiliations:** 1 Department of Medicine, University of Colorado Anschutz Medical Campus, Aurora; 2 Colorado Center for Transplantation Care, Research, and Education, University of Colorado Anschutz Medical Campus, Aurora; 3 Department of Surgery, University of Colorado Anschutz Medical Campus, Aurora; 4 Lerner Research Institute, Cleveland Clinic, Cleveland, Ohio

## Abstract

**Importance:**

Despite the acceptance of living-donor liver transplant (LDLT) as a lifesaving procedure for end-stage liver disease, it remains underused in the United States. Quantification of lifetime survival benefit and the Model for End-stage Liver Disease incorporating sodium levels (MELD-Na) score range at which benefit outweighs risk in LDLT is necessary to demonstrate its safety and effectiveness.

**Objective:**

To assess the survival benefit, life-years saved, and the MELD-Na score at which that survival benefit was obtained for individuals who received an LDLT compared with that for individuals who remained on the wait list.

**Design, Setting, and Participants:**

This case-control study was a retrospective, secondary analysis of the Scientific Registry of Transplant Recipients database of 119 275 US liver transplant candidates and recipients from January 1, 2012, to September 2, 2021. Liver transplant candidates aged 18 years or older who were assigned to the wait list (N = 116 455) or received LDLT (N = 2820) were included. Patients listed for retransplant or multiorgan transplant and those with prior kidney or liver transplants were excluded.

**Exposures:**

Living-donor liver transplant vs remaining on the wait list.

**Main Outcomes and Measures:**

The primary outcome of this study was life-years saved from receiving an LDLT. Secondary outcomes included 1-year relative mortality and risk, time to equal risk, time to equal survival, and the MELD-Na score at which that survival benefit was obtained for individuals who received an LDLT compared with that for individuals who remained on the wait list. MELD-Na score ranges from 6 to 40 and is well correlated with short-term survival. Higher MELD-Na scores (>20) are associated with an increased risk of death.

**Results:**

The mean (SD) age of the 119 275 study participants was 55.1 (11.2) years, 63% were male, 0.9% were American Indian or Alaska Native, 4.3% were Asian, 8.2% were Black or African American, 15.8% were Hispanic or Latino, 0.2% were Native Hawaiian or Other Pacific Islander, and 70.2% were White. Mortality risk and survival models confirmed a significant survival benefit for patients receiving an LDLT who had a MELD-Na score of 11 or higher (adjusted hazard ratio, 0.64 [95% CI, 0.47-0.88]; *P* = .006). Living-donor liver transplant recipients gained an additional 13 to 17 life-years compared with patients who never received an LDLT.

**Conclusions and Relevance:**

An LDLT is associated with a substantial survival benefit to patients with end-stage liver disease even at MELD-Na scores as low as 11. The findings of this study suggest that the life-years gained are comparable to or greater than those conferred by any other lifesaving procedure or by a deceased-donor liver transplant. This study’s findings challenge current perceptions regarding when LDLT survival benefit occurs.

## Key Points

**Question:**

Compared with the wait list, what is the survival benefit of a living-donor liver transplant for patients with end-stage liver disease based on patients’ Model for End-stage Liver Disease incorporating sodium levels (MELD-Na) score?

**Findings:**

In this case-control study of 119 275 liver transplant candidates, patients with a MELD-Na score as low as 11 had a 34% decrease in mortality compared with those who remained on the wait list. Patients who received a living-donor liver transplant can expect to gain an additional 13 to 17 years of life compared with those who never received a transplant.

**Meaning:**

This study’s findings challenge current perceptions regarding when the survival benefit of a living-donor transplant occurs.

## Teaser

This case-control study assesses the survival benefit, life-years saved, and the Model for End-stage Liver Disease incorporating sodium levels score at which that survival benefit was obtained for individuals who received a living-donor liver transplant compared with that for individuals who remained on the wait list.

## Introduction

Liver transplant is a life-saving procedure.^1^ The survival benefit has been established for deceased-donor liver transplant for patients with end-stage liver disease at a Model for End-stage Liver Disease incorporating sodium levels (MELD-Na) score^2^ of 15 or higher.^3^ However, each year, nearly 20% of patients awaiting a liver transplant in the United States die or become too sick for the transplant,^4^ demonstrating a severe shortage of donors and the dire necessity to increase the donor pool.

Given current allocation in the United States, patients with low MELD-Na scores (<15) rarely receive livers from deceased donors, yet these patients constitute the majority of new candidates added to the wait list.^4^ Patients with low MELD-Na scores must rely on either living donors or expanded-criteria deceased donors if they are to receive a transplant. However, the number of living-donor liver transplants (LDLTs) has scarcely increased during the past 20 years and still accounts for only 5% of liver transplants in the United States.^4^ The decision to use an LDLT involves weighing the risks of hepatectomy to the potential donor with the benefits to the recipient. Although donor risk is defined,^5,6,7^ part of the stagnation may stem from a lack of adequately powered studies providing clear quantification of recipient survival benefits and life-years saved with an LDLT, particularly at lower MELD-Na scores. In a subanalysis of the Adult-to-Adult Living Donor Liver Transplantation Cohort Study (A2ALL), the landmark multicenter living-donor consortium, the survival benefit of an LDLT was suggested at MELD-Na scores less than 15.^8^ However, this subanalysis was not powered to characterize a MELD-Na cutoff at which an LDLT provides more benefit than risk, nor did it quantify lifetime survival benefit. Therefore, to date, patients with low MELD-Na scores continue to experience a lack of accurate and consistent guidance concerning the ideal timing of an LDLT.

To address this need, we analyzed the Scientific Registry of Transplant Recipients (SRTR) database of liver transplant candidates and recipients from January 1, 2012, to September 2, 2021, to assess the survival benefit, life-years saved, and the MELD-Na score at which that survival benefit was obtained compared with those who remained on the wait list.

## Methods

### Data Sources

This case-control study used data from the SRTR. The SRTR data system includes data on all donors, waitlisted candidates, and transplant recipients in the United States, submitted by the members of the Organ Procurement and Transplantation Network.

### Study Population

The study population (N = 119 275) included patients aged 18 years or older who were assigned to the wait list (n = 116 455) or received a living-donor transplant (n = 2820) between January 1, 2012, and September 2, 2021. Patients listed for retransplant or multiorgan transplant were excluded, as were patients with prior kidney or liver transplants. eFigure 1 in the Supplement provides a population workflow diagram with the number of patients retained for each exclusion criterion. For patients listed before use of the MELD-Na score, MELD scores were recalculated to include sodium.^9^ We recalculated MELD-Na to include sodium for all patients listed on or after January 1, 2016, or a total of 73 196 waitlisted patients and 1891 patients receiving an LDLT, respectively. The biochemical MELD-Na score without exception points was used for the analysis. The race and ethnicity of the study participants were defined and recorded by the SRTR. The study was reviewed by an ethical committee (Colorado Multiple Institutions Review Board) and was determined to be nonhuman participants research, with a waiver of informed patient consent.

### Statistical Analysis

All study participants were stratified by MELD-Na scores at 6 to 10, 11 to 13, 14 to 16, 17 to 19, and 20 to 26 to provide a consistent representation of relative mortality, risk, and survival across MELD score ranges. All MELD score ranges other than 20 to 26 were adequately powered for all subsequent analyses. As such, a MELD score from 20 to 26 was excluded from the analysis of life-years from transplant.^10^ Survival times for waitlisted candidates started for all patients at the date of listing and were censored at the date of death or on removal from the wait list. Survival times for transplant recipients started at the date of transplant and were censored at the date of death or last follow-up. The mortality rate was calculated by dividing the number of deaths by the total patient-years and was reported as the rate of death per 1000 patient-years. Unadjusted hazard ratios were calculated by dividing the mortality rate of patients receiving an LDLT by that of waitlisted candidates. Adjusted hazard ratios were calculated with Cox proportional hazard regression analysis and adjusted for age at listing, sex, and primary diagnosis.^11^ The Cox proportional hazard model is a semiparametric regression method that models the association between survival time and 1 or more variables or covariates. Patients receiving a deceased-donor liver transplant were analyzed identically to those receiving an LDLT. All survival probability curves were generated with the nonparametric Kaplan-Meier estimation.^12^ Time to equal risk was reported as the day at which transplant survival intersected the probability of wait list survival. Time to equal survival was reported as the day at which the cumulative areas under the LDLT and waitlisted curves were equal.^13^ Life-years from transplant^10^ was calculated with parametric survival regression assuming a log-normal distribution and extrapolated to 10 000 days, or 27.38 years.^14^ Survival benefit in life-years was calculated by subtracting the median number of days on the wait list from life-years from transplant. Any participant without a listing date or MELD-Na score was removed from all analyses. All analyses were performed with the R statistical language, version 4.1.2 (R Core Team and the R Foundation for Statistical Computing).^15^ Throughout this study, we adhered to the Strengthening the Reporting of Observational Studies in Epidemiology (STROBE) reporting guideline.

## Results

The mean (SD) age of the 119 275 study participants was 55.1 (11.2) years, 75 112 (63%) were male, 1089 (0.9%) were American Indian or Alaska Native, 5097 (4.3%) were Asian, 9725 (8.2%) were Black or African American, 18 838 (15.8%) were Hispanic or Latino, 220 (0.2%) were Native Hawaiian or Other Pacific Islander, and 83 714 (70.2%) were White. Compared with patients on the wait list, recipients of an LDLT were younger (mean [SD] age, 53.0 [13.2] years vs 55.2 [11.1] years), more often female (1315 of 2820 [46.6%] female in the LDLT group vs 42 848 of 116 455 [36.8%] female on the wait list; odds ratio, 0.63; 95% CI, 0.59-0.68; *P* < .001), more educated (more than high school education: LDLT, 1735 of 2820 [62%]; wait list, 59 147 of 116 455 [51%]), and composed of a greater proportion of White individuals (2269 [80.5%] vs 81 445 [69.9%]) (Table). A greater proportion of recipients of an LDLT had a primary etiology of nonalcoholic steatohepatitis (558 [19.8%] vs 18 458 [15.8%]) and cholestatic liver disease (680 [24.1%] vs 8608 [7.4%]) compared with patients who remained on the wait list. At wait list placement, one-third of candidates had a MELD-Na score of 14 or higher (eFigure 2A in the Supplement). The distribution of MELD-Na scores for recipients of an LDLT was similarly skewed to the right, whereas two-thirds of the population had a MELD-Na score of 17 or higher (eFigure 2B in the Supplement).

**Table.** Characteristics of Wait List Candidates and Patients Who Received an LDLT, 2012-2021

Characteristic	Patients, No. (%)			*P* value
	Wait list (n = 116 455)^a^	LDLT (n = 2820)	Total (N = 119 275)	
Age, y				
Mean (SD)	55.2 (11.1)	53.0 (13.2)	55.1 (11.2)	<.001
Median (range)	57.0 (18.0-82.0)	56.0 (18.0-77.0)	57.0 (18.0-82.0)	
Sex				
Female	42 848 (36.8)	1315 (46.6)	44 163 (37.0)	<.001
Male	73 607 (63.2)	1505 (53.4)	75 112 (63.0)	
Race				
American Indian or Alaska Native	1076 (0.9)	13 (0.5)	1089 (0.9)	<.001
Asian	5014 (4.3)	83 (2.9)	5097 (4.3)	
Black or African American	9635 (8.3)	90 (3.2)	9725 (8.2)	
Hispanic or Latino	18 491 (15.9)	347 (12.3)	18 838 (15.8)	
Native Hawaiian or Other Pacific Islander	215 (0.2)	(0.2)^b^	220 (0.2)	
White	81 445 (69.9)	2269 (80.5)	83 714 (70.2)	
Missing	579 (0.5)	13 (0.5)	592 (0.5)	
Ethnicity				
Latino	18 648 (16.0)	349 (12.4)	18 997 (15.9)	<.001
Non-Latino or unknown	97 807 (84.0)	2471 (87.6)	100 278 (84.1)	
Education				
High school (grades 9-12)	45 301 (38.9)	852 (30.2)	46 153 (38.7)	<.001
Attended college or technical school	28 619 (24.6)	650 (23.0)	29 269 (24.5)	
Associate or bachelor degree	21 784 (18.7)	724 (25.7)	22 508 (18.9)	
Postcollege graduate degree	8744 (7.5)	361 (12.8)	9105 (7.6)	
Grade school (grades 0-8)	6060 (5.2)	101 (3.6)	6161 (5.2)	
None	391 (0.3)	7 (0.2)	398 (0.3)	
Missing	5556 (4.8)	125 (4.4)	5681 (4.8)	
Primary etiology				
Alcoholic cirrhosis	27 920 (24.0)	432 (15.3)	28 352 (23.8)	<.001
NASH cirrhosis	18 458 (15.8)	558 (19.8)	19 016 (15.9)	
Hepatitis C cirrhosis	4018 (3.5)	24 (0.9)	4042 (3.4)	
Hepatitis B cirrhosis	1908 (1.6)	29 (1.0)	1937 (1.6)	
Hepatocellular carcinoma	13 167 (11.3)	231 (8.2)	13 398 (11.2)	
Non-HCC malignancy	948 (0.9)	79 (2.8)	1027 (0.9)	
Cholestatic liver disease (PSC, PBC, or BA)	8608 (7.4)	680 (24.1)	9288 (7.8)	
Noncholestatic cirrhosis (other)	15 082 (13.0)	399 (14.1)	15 481 (13.0)	
Other	25 691 (22.1)	382 (13.5)	26 073 (21.9)	
Missing	655 (0.6)	6 (0.2)	661 (0.6)	

Abbreviations: BA, biliary atresia; HCC, hepatocellular carcinoma; LDLT, living-donor liver transplant; NASH, nonalcoholic steatohepatitis; PBC, primary biliary cholangitis; PSC, primary sclerosing cholangitis.

^a^ All patients on the wait list (excluding the individuals who received an LDLT) at the listing.

^b^ The number of patients was less than 10 and therefore not reported for privacy reasons.

**Figure 1.**
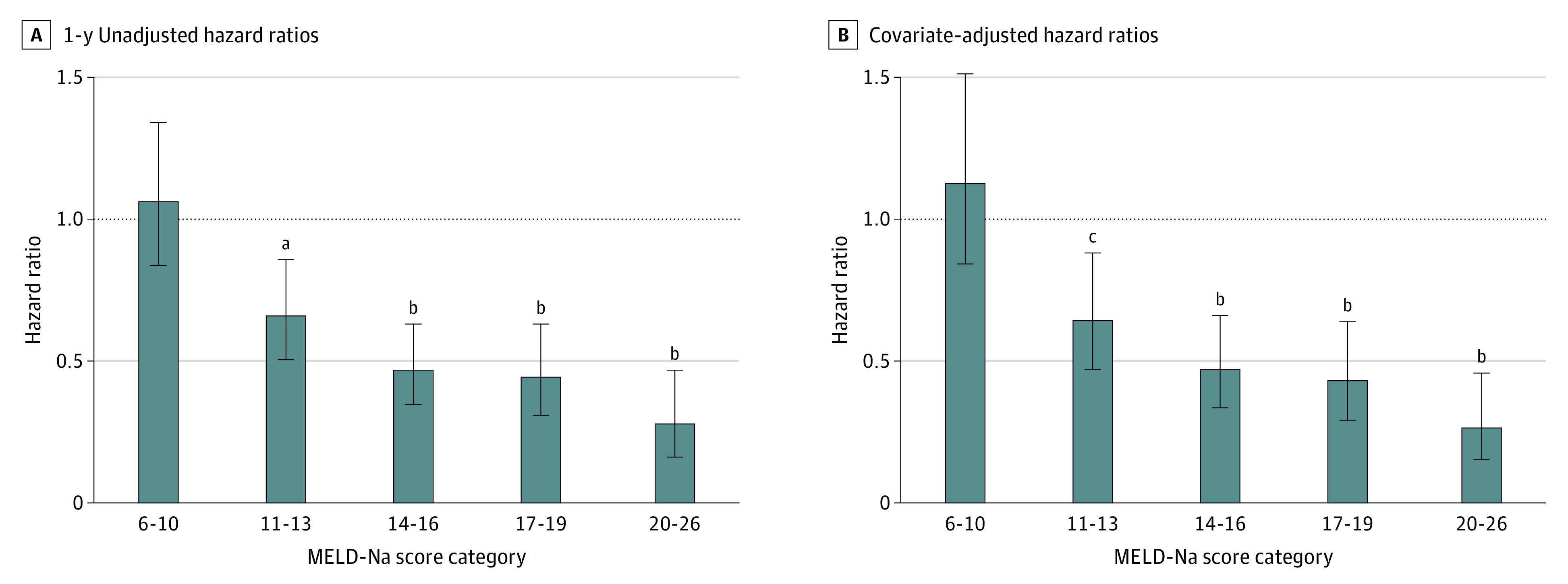
One-Year Mortality Risk Across Model for End-stage Liver Disease Incorporating Sodium Levels (MELD-Na) Score Categories for Patients Receiving a Living-Donor Liver Transplant vs Remaining on the Wait List, 2011-2021 Relative hazard ratios were calculated at 1 year on the wait list and after transplant across 5 MELD categories (scores 6-10, 11-13, 14-16, 17-19, and 20-26). One-year, unadjusted hazard ratios (A) and covariate-adjusted Cox proportional hazard ratios (B) were reported with 95% CIs and significance thresholds. Unadjusted hazard ratios were calculated by dividing the mortality rate of patients receiving a transplant by the mortality rate of waitlisted candidates. Adjusted hazard ratios were calculated with the Cox proportional hazard model and were adjusted for age at listing, sex, and primary diagnosis. ^a^*P* < .01. ^b^*P* < .05. ^c^*P* < .001.

A similar mortality rate existed between wait list candidates (56 deaths per 1000 patient-years) and recipients of an LDLT (60 deaths per 1000 patient-years) for very low MELD-Na scores (6-10) (eTable 1 in the Supplement), whereas the mortality rate was significantly less (between 34% and 72%) for all higher scores in MELD-Na groups. The survival benefit of LDLT was significant at a MELD-Na score as low as 11, with a 34% (95% CI, 17.4%-52.0%) decrease in mortality compared with the wait list. Unadjusted (Figure 1A) and covariate-adjusted (Figure 1B) mortality risk models confirmed the survival benefit of an LDLT for patients with a MELD-Na score of 11 or higher (MELD-Na scores 11-13: adjusted hazard ratio, 0.64 [95% CI, 0.47-0.88]; *P* = .006) (Figure 1B; eTable 2 in the Supplement) at 1 year after transplant. At a MELD-Na score of 14 to 16, mortality decreased by approximately 50% (hazard ratio, 0.47 [95% CI, 0.34-0.66]; *P* < .001) (Figure 1B; eTable 2 in the Supplement), and the benefit of an LDLT was associated with an increase in MELD-Na scores of 20 to 26 (Figure 1). For comparison, the risk of mortality was assessed for patients who received a deceased-donor liver transplant across the same MELD-Na categories (eFigure 3 in the Supplement). A consistent pattern of decreased risk for recipients of deceased-donor liver transplant was observed starting at MELD-Na scores of 11 to 13 (hazard ratio, 0.76 [95% CI, 0.69-0.84]; *P* = .006).

**Figure 2.**
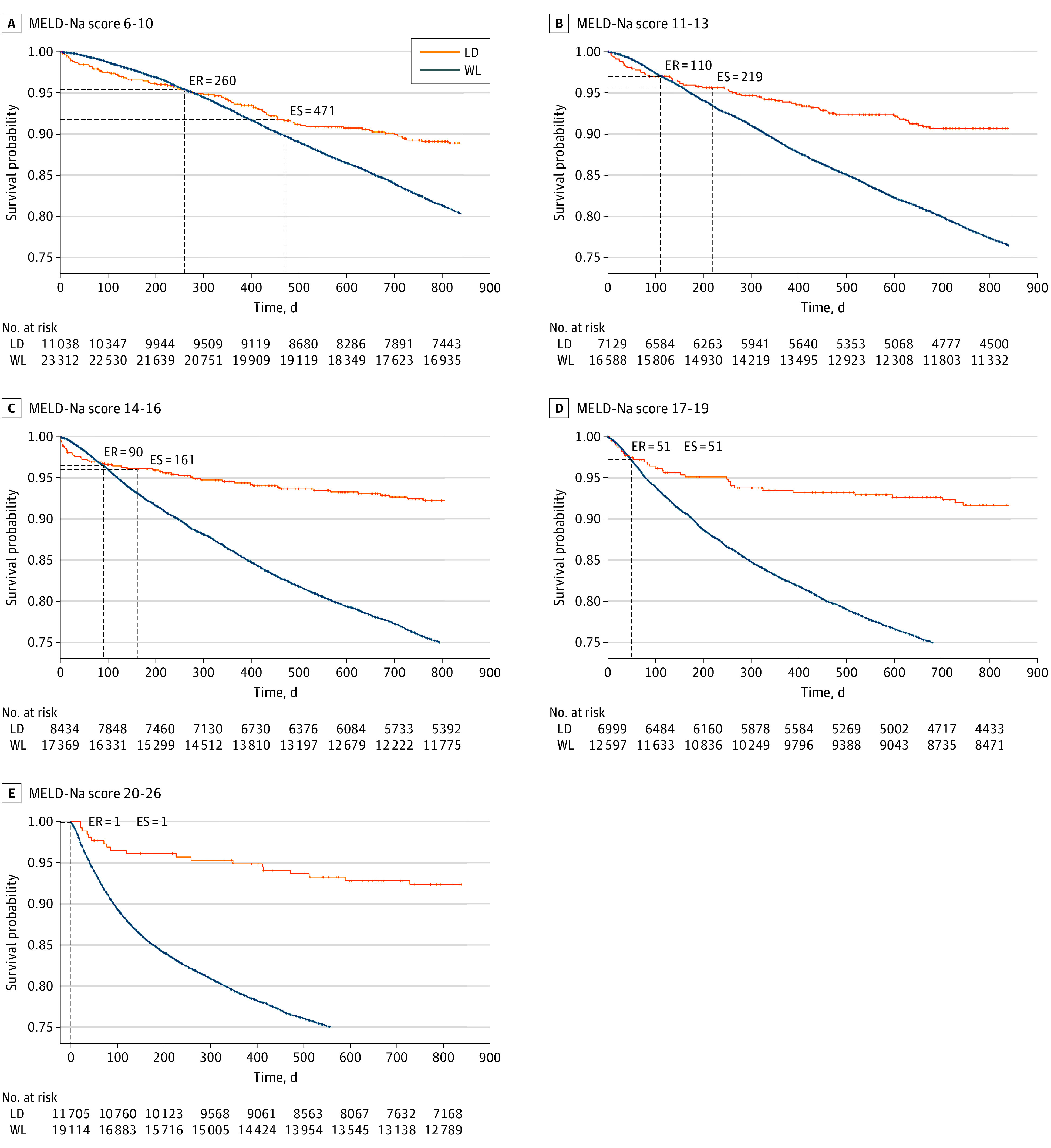
Survival Advantage of Living-Donor Liver Transplant (LDLT) vs Remaining on the Wait List Across 5 Model for End-stage Liver Disease Incorporating Sodium Levels (MELD-Na) Score Categories Survival probability curves were calculated for waitlisted candidates (WL) and patients receiving an LDLT (LD) across 5 MELD score categories with the nonparametric Kaplan-Meier estimation. Time to equal risk (ER) was reported as the day at which transplant survival probability intersected wait list survival probability. Time to equal survival (ES) was reported as the day at which the cumulative areas under the curves were equal. All LDLT survival curves were statistically significant (*P* < .001) compared with those for the wait list.

**Figure 3.**
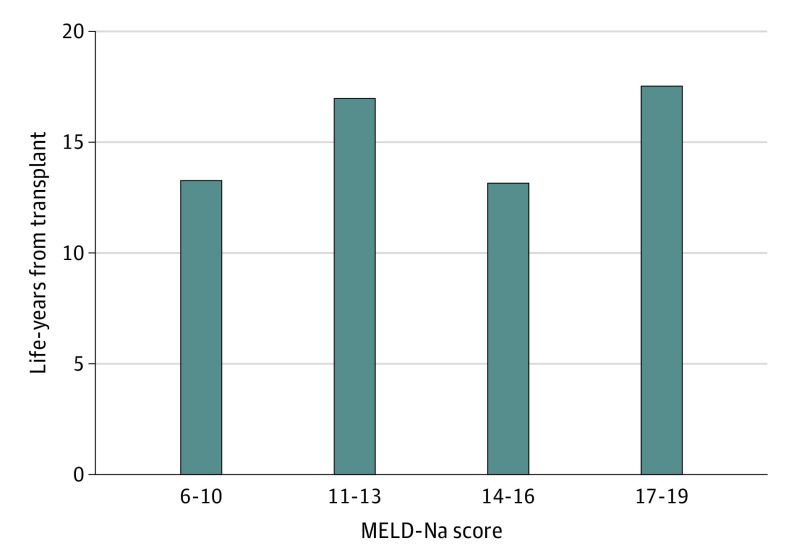
Life-Years Saved After Living-Donor Liver Transplant Life-years from transplant (LYFT) were calculated for Model for End-stage Liver Disease incorporating sodium levels (MELD-Na) groups with scores below 20 using parametric survival regression and extrapolated to 10 000 days, or 27.38 years. The MELD-Na score category of 20 to 26 was excluded from this analysis because this group was underpowered. The overall projected survival benefit, or life-years saved, was calculated by subtracting the median number of days on the wait list from life-years from transplant. The median life-years saved ranged from 13.2 to 17.6 years.

The probability of death from an LDLT for patients with very low MELD-Na scores (6-10) was greater than that for patients on the wait list for the first 259 days, at which point the risk of death for both groups was equal (time to equal risk); at 471 days, the probability of survival in both groups was equal (time to equal survival) (Figure 2A). As the MELD-Na score increased, the time to equal risk of death decreased (MELD-Na scores 11-13 = 110 days [Figure 2B]; MELD-Na scores 4-16 = 90 days [Figure 2C]; MELD-Na scores 17-19 = 51 days [Figure 2D]; and MELD-Na scores 20-26 = 1 day [Figure 2E]). The time to equal survival for patients on the wait list and those who received an LDLT also decreased as the MELD-Na score increased (MELD-Na scores 11-13 = 219 days [Figure 2B]; MELD-Na scores 14-16 = 161 days [Figure 2C]; MELD-Na scores 17-19 = 51 days [Figure 2D]; and MELD-Na scores 20-26 = 1 day [Figure 2E]), demonstrating that the survival benefit of an LDLT occurs much earlier for patients with a higher MELD-Na score. The survival benefit during a lifetime or life-years from transplant (Figure 3; eTable 3 in the Supplement) for patients who received an LDLT, even at very low MELD-Na scores, was substantial compared with remaining on the wait list and ranged from 13 to 17 additional years saved.

## Discussion

We present evidence from what is to our knowledge the largest study to date that shows the significant survival benefit of an LDLT for patients with end-stage liver disease and a MELD-Na score as low as 11, with a 34% decrease in mortality compared with that for patients on the wait list. Analysis of life-years from transplant showed that patients receiving an LDLT can expect to gain an additional 13 to 17 years of life compared with patients who never received a transplant. This survival benefit, particularly at low MELD-Na scores, is remarkable because previous studies with deceased donors argued that the benefit of a transplant occurs at MELD-Na scores of 15 or higher.^3^ Previous studies preceded the use of MELD-Na scores and direct-acting antivirals for chronic hepatitis C, which acted as a deterrent to an LDLT and suggested that patients with lower MELD-Na scores had relatively more risk than benefit. In fact, the survival benefit of a liver transplant at low MELD-Na scores (<15) is equivalent to or greater than that of other lifesaving procedures.

Patients with MELD-Na scores below 15 are significantly disadvantaged on the wait list, given current allocation policy.^4,16,17^ Thus, an LDLT may be their only option for receiving a liver transplant. Many in the transplant community have questioned the risks and benefits of a transplant for patients with lower MELD-Na scores, particularly given previous studies with deceased donors.^3^ This study’s findings definitively demonstrate the association of a marked benefit in survival and life-years with receipt of an LDLT. This association challenges the current paradigm of the timing of referral for a liver transplant and may have ramifications for allocation policies for deceased donors. These data also serve to inform potential donors of the benefit to their recipient to contextualize risk-benefit discussions.

An LDLT differs from a deceased-donor liver transplant in some essential respects. The former involves implantation of a partial graft as opposed to a full graft; there may be more constraints on recipient candidates according to size matching for an adequate graft-recipient weight ratio, and an LDLT is more susceptible to biliary stricture.^18,19^ Unfortunately, thus far, there has been a paucity of data to adequately demonstrate the potential survival advantages of an LDLT across the range of MELD-Na scores. Therefore, clinical care has relied on data from deceased-donor liver transplants to inform practice, despite the few studies that strongly suggest a survival benefit of an LDLT at MELD-Na scores below 15.^8,20,21^ The present study is therefore timely and conclusively challenges current perceptions regarding the MELD-Na score threshold at which a survival benefit is derived.

### Strengths and Limitations

The strengths of this study are that it is adequately powered at MELD-Na scores at which patients most commonly receive an LDLT, the time frame chosen coincides with the maturation of the LDLT experience in the United States, and there are adequate numbers to evaluate different eras of predominant indications for a liver transplant. Small studies have measured the survival benefit of an LDLT^8^; however, to our knowledge, none have examined the lifetime survival benefit or have been adequately powered to investigate the full spectrum of patients with lower MELD-Na scores. To our knowledge, the present study is the first to show a significant benefit in life-years saved over a lifetime following an LDLT.

Still, these data must be interpreted within the confines of the study limitations. First, the number of patients with high MELD-Na scores (>26) who received an LDLT was relatively small owing to US practice patterns of using an LDLT for patients with lower MELD-Na scores. Therefore, this study was not fully powered to provide an interpretation of the survival benefits of an LDLT for patients with MELD-Na scores higher than 26, although the trend in survival benefit persisted. Similarly, the study was not powered to assess the survival advantage based on the etiology of end-stage liver disease; however, we addressed this shortcoming by adjusting models for etiology. As in all retrospective registry studies, one cannot exclude nonrandom selection bias. This selection bias likely involved both those patients who were “sicker” than their MELD score would indicate and those “healthier” than their MELD score would indicate, and thus it would not materially change the expected life-year benefit. To address the potential association between the changing etiologies of end-stage liver disease and the indication for a liver transplant (eTable 4 in the Supplement), we evaluated the survival benefit for transplant candidates before and after 2016 to reflect the association between direct-acting antiviral therapy to cure chronic hepatitis C and implementation of MELD-Na scores.^22^ We found comparable benefits throughout all MELD-Na scores, confirming the generalizability of our findings across these distinct eras.

## Conclusions

As performed in the United States, an LDLT confers a substantial survival benefit to patients with end-stage liver disease even at MELD-Na scores as low as 11. This benefit is associated with increasing MELD scores. The life-years gained are comparable to or greater than those of any other lifesaving procedure. This study’s findings challenge current perceptions regarding when a liver transplant survival benefit occurs. Our results suggest that, for patients with MELD-Na scores higher than 11, nationwide acceptance of an LDLT as a superior alternative to waiting for a deceased donor will significantly increase survival compared with remaining on the wait list.

## Notes

Supplement. **eFigure 1.** Study Population Workflow, 2012-2101 **eFigure 2.** MELD-Na Score Distributions for Waitlisted Candidates and Live-Donor Liver Transplant Recipients **eFigure 3.** One-Year Mortality Risk Across MELD-Na Categories for Patients Receiving Deceased-Donor Liver Transplantation (DDLT) vs Remaining on the Waitlist, 2011-2021 **eTable 1.** Mortality Rate for Patients on the Waitlist vs LDLT **eTable 2.** Risk of Mortality for Patients on the Waitlist vs LDLT **eTable 3.** Life-Years Saved After LDLT **eTable 4.** Patient Characteristics in Cohorts, 2012-2015 and 2016-2021
