# Supplementary material for: Survival Benefit of Living-Donor Liver Transplant
Source: JAMA Surg. 2022 Aug 3;157(10):926–32. doi: 10.1001/jamasurg.2022.3327 (PMC9350845; doi:10.1001/jamasurg.2022.3327)
Supplement: Supplement. — eFigure 1. Study Population Workflow, 2012-2101 eFigure 2. MELD-Na Score Distributions for Waitlisted Candidates and Live-Donor Liver Transplant Recipients eFigure 3. One-Year Mortality Risk Across MELD-Na Categories for Patients Receiving Deceased-Donor Liver Transplantation (DDLT) vs Remaining on the Waitlist, 2011-2021 eTable 1. Mortality Rate for Patients on the Waitlist vs LDLT eTable 2. Risk of Mortality for Patients on the Waitlist vs LDLT eTable 3. Life-Years Saved After LDLT eTable 4. Patient Characteristics in Cohorts, 2012-2015 and 2016-2021 [file jamasurg-e223327-s001.pdf]

Supplemental Online Content

Jackson WE, Malamon JS, Kaplan B, et al. Survival benefit of living-donor liver transplant. *JAMA Surg*. Published online August 3, 2022.
doi:10.1001/jamasurg.2022.3327

eFigure 1. Study Population Workflow, 2012-2101
eFigure 2. MELD-Na Score Distributions for Waitlisted Candidates and Live-Donor Liver Transplant Recipients
eFigure 3. One-Year Mortality Risk Across MELD-Na Categories for Patients Receiving Deceased-Donor Liver Transplantation (DDLT) vs Remaining on the Waitlist, 2011-2021
eTable 1. Mortality Rate for Patients on the Waitlist vs LDLT
eTable 2. Risk of Mortality for Patients on the Waitlist vs LDLT
eTable 3. Life-Years Saved After LDLT
eTable 4. Patient Characteristics in Cohorts, 2012-2015 and 2016-2021

This supplemental material has been provided by the authors to give readers additional information about their work.

© 2022 Jackson WE et al. *JAMA Surgery*.

eFigure 1. Study Population Workflow, 2012-2021

The number of patients for each cohort, waitlisted (WL), living donor (LD), and deceased donor (LD), are provided in the columns (boxes) above. Each row provides the total number of patients per cohort after filtering. Patients were filtered based on age greater than 17 years, placement or transplant date on or after January 1, 2012, re-transplantation status, missing MELD score and placement date.

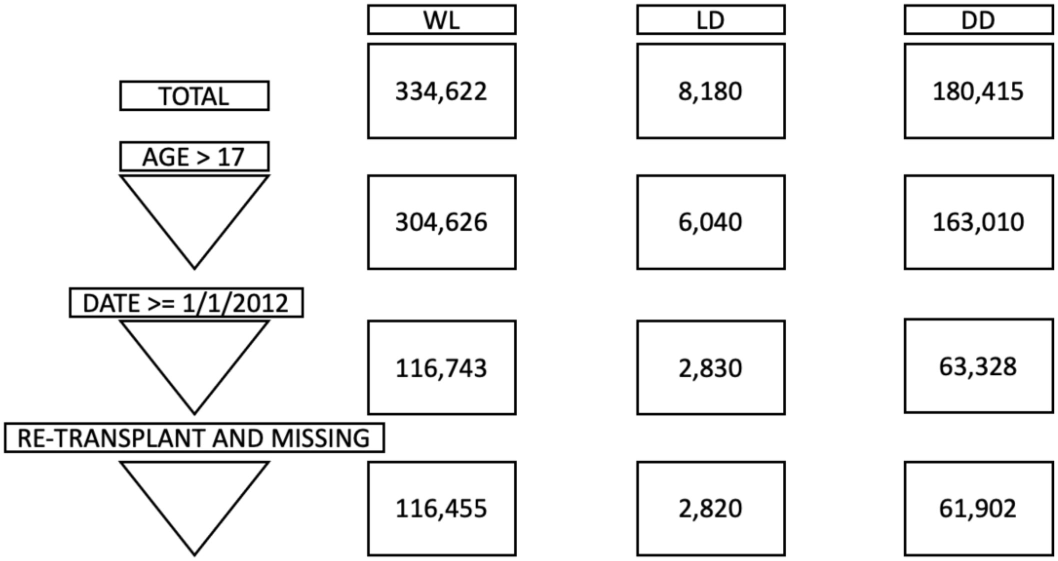

© 2022 Jackson WE et al. *JAMA Surgery*.

eFigure 2. MELD-Na Score Distributions for Waitlisted Candidates and Live-Donor Liver Transplant Recipients

MELD-Na score distributions are provided at initial placement on waitlist for A) waitlisted candidates and B) at the time of transplant for patients receiving a live donor liver transplant (LDLT). ~55% of waitlisted candidates had a MELD-Na equal to or greater than 20. ~82% of LDLT patients had a MELD-Na equal to or greater than 20 at placement on the waitlist.

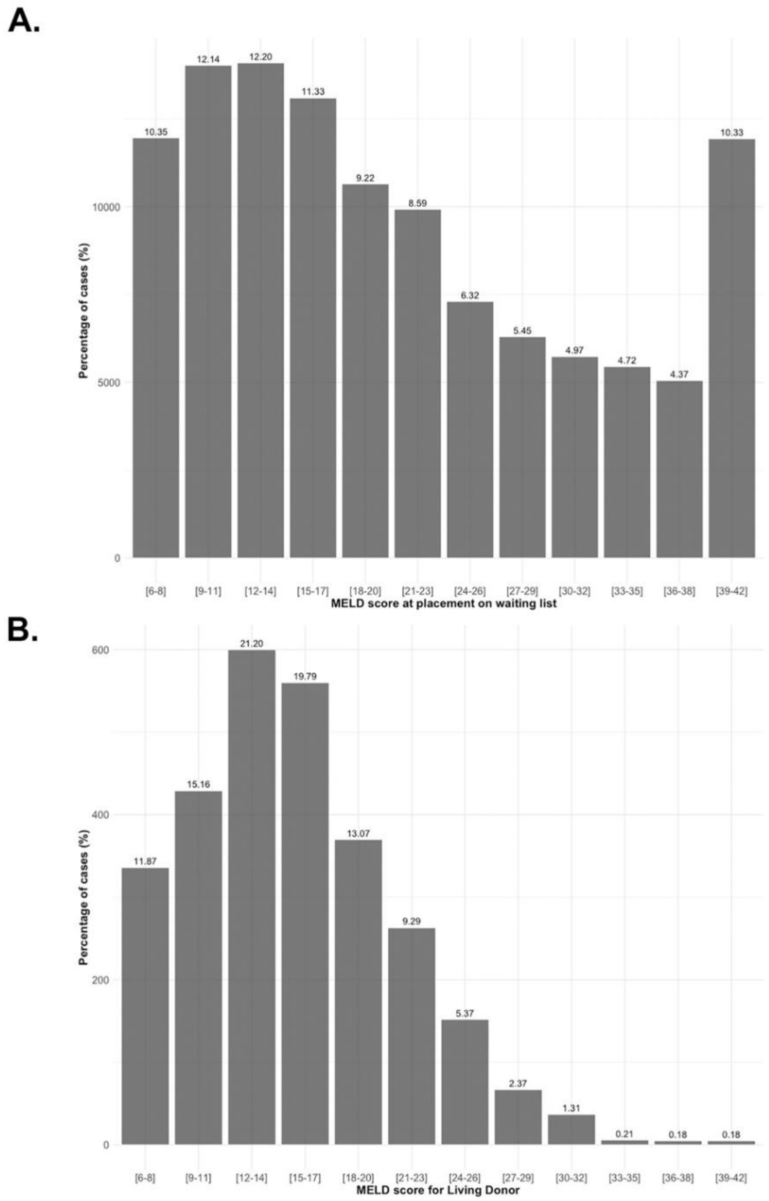

© 2022 Jackson WE et al. *JAMA Surgery*.

eFigure 3. One-Year Mortality Risk Across MELD-Na Categories for Patients Receiving Deceased-Donor Liver Transplantation (DDLT) vs Remaining on the Waitlist, 2011-2021

Relative hazard ratios were calculated at one-year on the waitlist and post-transplantation for deceased donor liver transplants (DDLT) across eight MELD-Na categories: 6-10, 11-13, 14-16, 17-19, 20-26, 27-30, 31-38, and 39>. A) one-year, unadjusted hazard ratios and B) covariate-adjusted Cox proportional hazard ratios were reported with 95% confidence intervals and significance thresholds. Unadjusted hazard ratios were calculated by diving the mortality rate of DDLT patients by the mortality rate of waitlisted candidates. Adjusted hazard ratios were calculated using the Cox proportional hazard model and were adjusted for age at listing, sex, and primary diagnosis. Significance thresholds were labelled as: *p<0.05, **p<0.01, and ***p<0.001.

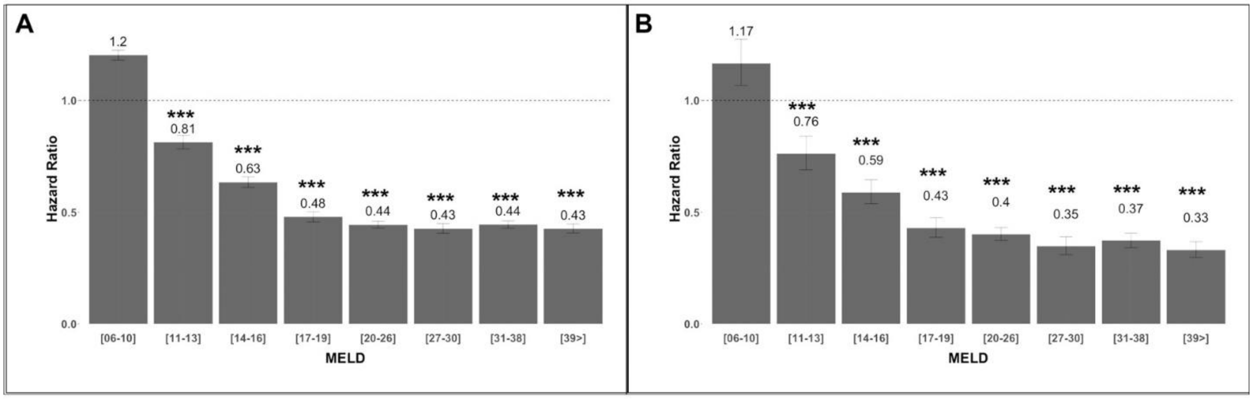

© 2022 Jackson WE et al. *JAMA Surgery*.

eTable 1. Mortality Rate for Patients on the Waitlist vs LDLT

	Waitlist			LDLT		
**MELD-Na**	**Deaths**	**PY**	**Rate/1000 PY**	**Deaths**	**PY**	**Rate/1000 PY**
[06-10]	1401	25024	55.9863	47	788	59.6447
[11-13]	1643	18226	90.1459	40	671	59.6125
[14-16]	2144	19053	112.5282	34	644	52.795
[17-19]	1968	14263	137.9794	25	407	61.4251
[20-26]	3615	20874	173.1819	13	268	48.5075

MELD = model for end-stage liver disease, PY = patient years, LDLT = living donor liver transplant

© 2022 Jackson WE et al. *JAMA Surgery*.

eTable 2. Risk of Mortality for Patients on the Waitlist vs LDLT

	Unadjusted			Adjusted		
**MELD-Na**	**HR**	**CI**	**P-Value**	**HR**	**CI**	**P-Value**
[06-10]	1.07	(0.84 - 1.34)	0.678	1.13	(0.84 - 1.51)	0.415
[11-13]	0.66	(0.50 - 0.86)	0.013	0.64	(0.47 - 0.88)	0.006
[14-16]	0.47	(0.35 - 0.63)	<0.001	0.47	(0.34 - 0.66)	<0.001
[17-19]	0.45	(0.31 - 0.63)	<0.001	0.43	(0.29 - 0.64)	<0.001
[20-26]	0.28	(0.16 - 0.47)	<0.001	0.27	(0.15 - 0.46)	<0.001

MELD = model for end-stage liver disease, HR = hazard ratio, LDLT = living donor liver transplant

© 2022 Jackson WE et al. *JAMA Surgery*.

eTable 3. Life-Years Saved After LDLT

MELD	Median Waitlist Days	Median Waitlist Years	LYFT	Net Years Gained	P-Value
[06-10]	1966	5.383	18.829	13.447	<0.001
[11-13]	1886	5.164	21.953	16.789	<0.001
[14-16]	1844	5.049	18.201	13.152	<0.001
[17-19]	1729	4.734	22.414	17.681	<0.001

MELD = model for end-stage liver disease, LYFT = life years saved following transplant

© 2022 Jackson WE et al. *JAMA Surgery*.

eTable 4. Patient Characteristics in Cohorts, 2012-2015 and 2016-2021

	2012-2015				2016-2021			
	**W (N=45150)**	**L (N=929)**	**Total (N=46079)**	**P-value**	**W (N=71305)**	**L (N=1891)**	**Total (N=73196)**	**P-value**
**Age**								
Mean (SD)	55.1 (10.5)	52.3 (12.9)	55.1 (10.6)	<0.001	55.2 (11.5)	53.3 (13.3)	55.1 (11.5)	<0.001
Median [Min, Max]	57.0 [18.0, 81.0]	55.0 [18.0, 75.0]	57.0 [18.0, 81.0]		58.0 [18.0, 82.0]	56.0 [18.0, 77.0]	58.0 [18.0, 82.0]	
**Sex**								
F	16144 (35.8%)	404 (43.5%)	16548 (35.9%)	<0.001	26704 (37.5%)	911 (48.2%)	27615 (37.7%)	<0.001
M	29006 (64.2%)	525 (56.5%)	29531 (64.1%)		44601 (62.5%)	980 (51.8%)	45581 (62.3%)	
**Race**								
Caucasian	31649 (70.1%)	760 (81.8%)	32409 (70.3%)	<0.001	49796 (69.8%)	1509 (79.8%)	51305 (70.1%)	<0.001
Hispanic/Latino	6684 (14.8%)	104 (11.2%)	6788 (14.7%)		11807 (16.6%)	243 (12.9%)	12050 (16.5%)	
Black or African American	4226 (9.4%)	28 (3.0%)	4254 (9.2%)		5409 (7.6%)	62 (3.3%)	5471 (7.5%)	
Asian	1984 (4.4%)	27 (2.9%)	2011 (4.4%)		3030 (4.2%)	56 (3.0%)	3086 (4.2%)	
American Indian or Alaska Native	350 (0.8%)	3 (0.3%)	353 (0.8%)		726 (1.0%)	10 (0.5%)	736 (1.0%)	
Native Hawaiian or Other Pacific Islander	83 (0.2%)	3 (0.3%)	86 (0.2%)		132 (0.2%)	2 (0.1%)	134 (0.2%)	
Missing	174 (0.4%)	4 (0.4%)	178 (0.4%)		405 (0.6%)	9 (0.5%)	414 (0.6%)	
Ethnicity								
Latino	6717 (14.9%)	104 (11.2%)	6821 (14.8%)	0.0075	11931 (16.7%)	245 (13.0%)	12176 (16.6%)	<0.001
Non-Latino or unknown	38433 (85.1%)	825 (88.8%)	39258 (85.2%)		59374 (83.3%)	1646 (87.0%)	61020 (83.4%)	
**Education**								
Associate/bachelor’s degree	7677 (17.0%)	249 (26.8%)	7926 (17.2%)	<0.001	14107 (19.8%)	475 (25.1%)	14582 (19.9%)	<0.001
Attended College/Technical School	11071 (24.5%)	197 (21.2%)	11268 (24.5%)		17548 (24.6%)	453 (24.0%)	18001 (24.6%)	
Grade School (0-8)	2238 (5.0%)	25 (2.7%)	2263 (4.9%)		3822 (5.4%)	76 (4.0%)	3898 (5.3%)	
High School (9-12)	18310 (40.6%)	305 (32.8%)	18615 (40.4%)		26991 (37.9%)	547 (28.9%)	27538 (37.6%)	
None	144 (0.3%)	4 (0.4%)	148 (0.3%)		247 (0.3%)	3 (0.2%)	250 (0.3%)	

© 2022 Jackson WE et al. *JAMA Surgery*.

Post-College Graduate Degree	3257 (7.2%)	121 (13.0%)	3378 (7.3%)		5487 (7.7%)	240 (12.7%)	5727 (7.8%)	
Missing	2453 (5.4%)	28 (3.0%)	2481 (5.4%)		3103 (4.4%)	97 (5.1%)	3200 (4.4%)	
**Primary Etiology**				<0.001				<0.001
Alcoholic cirrhosis	8085 (17.9%)	121 (13.0%)	8206 (17.8%)		19835 (27.8%)	311 (16.4%)	20146 (27.5%)	
NASH cirrhosis	5363 (11.9%)	122 (13.1%)	5485 (11.9%)		13095 (18.4%)	436 (23.1%)	13531 (18.5%)	
Hepatitis C cirrhosis	13591(30.1%)	199 (21.4%)	13790 (29.9%)		9335 (13.1%)	142 (7.5%)	9477 (12.9%)	
Hepatitis B cirrhosis	857 (1.9%)	11 (1.2%)	868 (1.9%)		1051 (1.5%)	18 (1.0%)	1069 (1.5%)	
Hepatocellular carcinoma	5047 (11.2%)	72 (7.8%)	5119 (11.1%)		8120 (11.4%)	159 (8.4%)	8279 (11.3%)	
Non-HCC malignancy	321 (0.8%)	25 (2.7%)	346 (0.9%)		627 (1.0%)	54 (2.9%)	681 (1.0%)	
Cholestatic Liver Disease (PSC, PBC, BA)	3286 (7.3%)	213 (22.8%)	3499 (7.5%)		5322 (7.4%)	467 (25.0%)	5789 (8.0%)	
Non-cholestatic cirrhosis (other)	6088 (13.2%)	142 (15.1%)	6230 (13.2%)		8994 (12.5%)	257 (13.8%)	9251 (12.5%)	
Missing	14 (0.0%)	1 (0.1%)	15 (0.0%)		641 (0.9%)	5 (0.3%)	646 (0.9%)	
Other	2498 (5.5%)	23 (2.5%)	2521 (5.5%)		4285 (6.0%)	42 (2.2%)	4327 (5.9%)	

NASH = non-alcoholic steatohepatitis; HCC = Hepatocellular carcinoma; PSC = Primary Sclerosing Cholangitis; PBC = primary biliary cholangitis; BA = Biliary atresia

© 2022 Jackson WE et al. *JAMA Surgery*.
